# The economic cost of implementing antigen-based rapid diagnostic tests for COVID-19 screening in high-risk transmission settings: evidence from Germany

**DOI:** 10.1186/s13561-022-00361-3

**Published:** 2022-02-14

**Authors:** Alfonso Valenzuela Hurtado, Hoa Thi Nguyen, Viktoria Schenkel, Jonas Wachinger, Joachim Seybold, Claudia M. Denkinger, Manuela De Allegri

**Affiliations:** 1 Heidelberg Institute of Global Health, Heidelberg University Hospital and Faculty of Medicine, Heidelberg University, Heidelberg, Germany; 2 Division of Clinical Tropical Medicine, Heidelberg University Hospital and Faculty of Medicine, Heidelberg University, Heidelberg, Germany; 3 Charité –Universitätsmedizin Berlin, corporate member of Freie Universität Berlin, Humboldt-Universität zu Berlin, and Berlin Institute of Health; Medical Directorate, Berlin, Germany

**Keywords:** Antigen-based rapid diagnostic test, SARS-CoV-2, Screening, Implementation costs, Germany

## Abstract

**Background:**

Antigen-based rapid diagnostic tests (Ag-RDT) have been implemented in hospitals and nursing homes to screen for infectious individuals without symptoms suggestive of SARS-CoV-2 infections and to prevent entry into these high-risk settings. Despite their benefits for screening, the cost of large-scale implementation is largely understudied. Our study presents evidence on their implementation costs in high-risk settings.

This study aimed to estimate the economic costs of implementing Ag-RDT-based screening for SARS-CoV-2 in two tertiary care hospitals (University Hospital Heidelberg - UKHD, and Charité – Universitätsmedizin Berlin) and one nursing home in Germany.

**Methods:**

We adopted a health system perspective and followed the three sequential steps to costing: identification of resources, measurement of resource consumption, and valuation of costs. Data on resource consumption were collected between October 2020 and April 2021 through various techniques and data sources. The cost estimation considered all costs along the screening algorithm including PCR confirmation tests for positive cases. We estimated the costs for the two implementation modalities observed: staff dedicated exclusively to screening and staff not dedicated exclusively to screening. Furthermore, cost estimations were performed under both observed capacity use and hypothetical capacity use assumptions (60, 80 and 100%).

**Results:**

Our study indicates that the average cost per Ag-RDT is highly dependent on the capacity use and implementation mode. Staff time and test kits are the two main cost drivers of implementing the large-scale screening programs for SARS-CoV-2 using Ag-RDTs. For hospitals, the average cost per test in UKHD was €30.12 (capacity observed); €14.56 (non-dedicated mode); €19.47, €16.37, €14.53 at 60, 80, 100% capacity respectively (dedicated mode); and at Charité €13.10 (non-dedicated mode). For the nursing home the estimated average cost per test was €15.03 (non-dedicated mode).

**Conclusions:**

The information on the estimated costs by mode of implementation and capacity use may support the planning of Ag-RDT-based covid-19 screening programs suitable for each institution. Further research is needed to cost this screening strategy for COVID-19 in other high-risk, high-income settings to reach generalizability.

## Introduction

Despite increasing vaccination rates for SARS-CoV-2, screening of asymptomatic individuals remains crucial, as they may account for up to 45% of all SARS-CoV-19 infections [1]. This is particularly important upon entry in settings with persons that are at high risk of severe COVID-19 infection, such as hospitals and nursing homes, where it is critical to avoid exposure to the virus and thereby avoid new infections [2, 3]. However, scaling-up screening has become a major challenge during this pandemic [4].

Among the different SARS-CoV-2 diagnostic tests approved by the WHO and national health authorities, the gold standard is the reverse transcriptase–polymerase chain reaction (RT-PCR) [5]. However, the turn-around time of large throughput RT-PCR often renders it ineffective for rapid detection [3], mainly in high-risk settings (e.g. hospitals or nursing homes), where screening for asymptomatic individuals is key to deciding on access and thus minimizing the exposure to the virus of the population at risk. Consequently, everyday practice requires complementary diagnostic alternatives to detect infectious individuals [6]. An easy-to-use, rapid point of care (PoC) test can be used to expand screening capacity [7]. SARS-CoV-2 antigen-based rapid detection tests (Ag-RDTs) offer good accuracy, rapid PoC detection of infectious individuals and ease of use [7]. Therefore, implementation of Ag-RTDs for screening asymptomatic individuals has occurred widely in Germany in nursing homes and to some extent in hospitals. However, no published data exist on the economic costs of the implementation of Ag-RDT-based screening programs for COVID-19 in high-risk settings.

In the absence of evidence on the costs of screening asymptomatic people with Ag-RDTs in high-risk settings, and in order to inform the roll-out of Ag-RDT-based screening programs, we estimated the costs of implementing Ag-RDT-based screening programs for asymptomatic persons in high-risk settings. Given the continued preponderance of the pandemic, our ambition has been that of generating a sound evidence-base to guide further implementation of screening in high-risk settings, considering the full economic cost of the program beyond only the cost of the single test kit. Specifically, we estimated the costs in two university hospitals and one nursing home in Germany and addressed three objectives: i) estimate the average cost per test implemented in hospital settings; ii) project the estimated cost for patient screening using Ag-RDTs in hospitals with different patient volumes; iii) estimate the average cost per test implemented in nursing homes.

## Methods

### Study setting and intervention

This study was carried out in two tertiary hospitals in Germany: Heidelberg University Hospital (UKHD) and Charité – Universitätsmedizin Berlin (Charité). UKHD and Charité are two of the largest tertiary hospitals in Germany, with 1991 [8] and 3001 [9] beds, respectively. Both institutions have large laboratory services capacity for patient diagnosis (including RT-PCR testing); however, laboratory capacity has been constrained due to increased demand since the onset of the current pandemic. Therefore, UKHD implemented Ag-RDTs for the screening of patients presenting for elective procedures without symptoms suggestive of SARS-CoV-2 (including day clinics) prior to admission in all clinics starting from October 2020, either by dedicated staff (staff already employed in the facility who are redirected to work exclusively on Ag-RTDs patient screening), or by non-dedicated staff (staff already employed in the facility who perform Ag-RDTs only when required within the framework of their daily work routine). Ag-RDTs at Charité were implemented for the screening of patients presenting for elective procedures without symptoms suggestive of SARS-CoV-2 in the neurology outpatient department from March to April 2021, using the non-dedicated staff mode only for the purpose of this study. Both hospitals used the Standard Q COVID-19 Ag kit (SD Biosensor, Republic of Korea) for patient screening due to its high sensitivity (76.6%) and specificity (99.3%) in persons with high viral load infections (CT < 25) [10]. In patients with a positive Ag-RDT, an additional nasopharyngeal swab was collected for confirmatory RT-PCR [11].

Separately, we assessed costs in a nursing home in the Rhein-Neckar-Kreis, an administrative district in the South of Germany. In this setting, screening was carried out only with non-dedicated staff for residents, visitors and health workers. The two implementation mode categories, dedicated staff and non-dedicated staff, reflect the different ways in which staff were engaged to carry out rapid testing in each of the settings, with different implications in terms of opportunity cost. In other words, dedicated staff is exclusively assigned to implement Ag-RDTs, and when the number of patients tested is lower than the planned testing volume, their time is unused and thus the opportunity cost of their time increase. Meanwhile, non-dedicated staff performs Ag-RDT testing alongside with their routine nursing tasks, thus we assumed that their time is used at 100% capacity and accordingly, the opportunity cost of their time is not affected by varying testing volume.

### Study design

This costing study adopted a health system perspective to provide relevant evidence to decision makers on the economic cost of implementing Ag-RDTs. Data on resource consumption were collected from all study sites between October 2020 and April 2021.

In line with the study objectives, we followed the bottom-up micro-costing approach [12] to estimate the cost of implementing Ag-RTDs in the two hospitals (study objective 1) and the nursing home (study objective 3). To project the costs associated with patient screening using Ag-RDTs in hospitals of different size in Germany (study objective 2), we compiled the average cost estimates obtained in study objective 1 with data on service volume for hospitals of different patient volume from open data sources.

### Costing procedures and data sources

Our micro-costing approach unfolded over three sequential steps. First, we identified which resources were necessary for the implementation of Ag-RDTs based on standard operating procedures (SOPs) and direct observation (only in hospitals). Subsequently, we classified the identified items in recurrent costs (staff, test kits and consumables, protective gear, and variable overheads) and capital costs (fix overheads, building, and equipment).

Second, we measured the consumption for the identified resources using different techniques and data sources. Table 1 presents an overview of the methods and data sources for the measurement of resource consumption and the valuation of costs used in all study settings. For recurrent costs, given that staff time to implement a test can vary, we conducted direct observation at UKHD and Charité to record the average time employed by staff to complete the different steps to perform a test, including patient registration, sample taking, test implementation, results monitoring and documentation. Since direct observation at the nursing home was not possible, we assumed that staff time consumption would be equivalent to what was observed at UKHD and Charité. We measured the resource consumption of test kits and other recurrent cost items using the corresponding SOPs for Ag-RDTs implementation from the respective study sites. The data from UKHD was utilized for the estimations of the nursing home.

**Table 1:** Methods and data sources of costs in all study settings

COST TYPE	ITEMS	IDENTIFIED ITEMS	MEASUREMENT	VALUATION	
			METHODS	METHODS	DATA SOURCE
**RECURRENT**	**STAFF**	Trainer	Direct observation	Bottom-up	Online - State average salary (BW)* / Charité internal data
		Nurses	Direct observation	Bottom-up and Top-down	Online - State average salary (BW)* / Charité internal data
		Receptionist	Direct observation	Bottom-up and Top-down	Online - State average salary (BW)* / Charité internal data
	**TEST KITS + CONSUMABLES**	Antigen test kit	SOP	Bottom-up	Charité internal price / Charité internal data
		Hand sanitizing solution	Estimated consumption per-test	Bottom-up	Online - Market price / Charité internal data
		Tissues	Estimated consumption per-test	Bottom-up	Online - Market price / Charité internal data
	**PROTECTIVE GEAR**	Face shield	SOP	Bottom-up	Online - Market price / Charité internal data
		Surgical gown	SOP	Bottom-up	Online - Market price / Charité internal data
		Scrub suit (pants + kasack)	SOP	Bottom-up	Online - Market price / Charité internal data
		Surgical cap	SOP	Bottom-up	Online - Market price / Charité internal data
		Face mask FFP2	SOP	Bottom-up	Online - Market price / Charité internal data
		Nitril gloves	SOP	Bottom-up	Online - Market price / Charité internal data
	**VARIABLE OVERHEADS**	Electricity	Secondary data	Top-down	Online - DESTATIS / Charité internal data
		Water	Secondary data	Top-down	Online - DESTATIS / Charité internal data
		Internet	Secondary data	Top-down	Online - DESTATIS / Charité internal data
		Telephone	Secondary data	Top-down	Online - DESTATIS / Charité internal data
		Medical waste disposal	Secondary data	Top-down	Online - DESTATIS / Charité internal data
**CAPITAL**	**FIX OVERHEADS**	Management/procurement	Secondary data	Top-down	Online - DESTATIS / Charité internal data
	**BUILDING**	Room/Space	Direct observation	Bottom-up	Quotation / Charité internal data
	**EQUIPMENT**	PC	Physical counting	Bottom-up	Online - Market price / Charité internal data
		ID Scanner	Physical counting	Bottom-up	Online - Market price / Charité internal data
		Label printer	Physical counting	Bottom-up	Online - Market price / Charité internal data

*BW: Baden-Württemberg

Third, to value costs of resource consumption we applied both a bottom-up and a top-down approach (Table 1). For recurrent costs, we valued staff cost considering the two different implementation modes, i.e., dedicated vs. non-dedicated staff. Specifically, for the non-dedicated staff mode of implementation (implemented in Charité, in certain UKHD clinics, and the nursing home), we estimated staff cost by multiplying the average time in minutes needed to perform one test by the staff cost per minute. For the dedicated staff mode (implemented in other UKHD clinics), we estimated the staff cost per test by dividing the daily staff cost by the number of tests performed daily. Since nurses carry out most tests in our study settings, we used the average cost of nurses to value health staff costs, which include gross salaries and costs to the employer. In addition, we added the corresponding cost of training to the staff cost per test. Given that test kits contain all the necessary materials to carry out one test, we used the purchase price of the test kit to value the cost of all test materials. Moreover, we added the cost of the consumables required per test (hand sanitizing solution and tissues). We valued the costs of protective gear by multiplying the quantities of single-use items and the estimated use of multiple-use items per test by the corresponding unit cost (bottom-up approach), obtained either from market prices for UKHD or internal information for Charité (Table 1). We valued variable overhead using estimates of hospital cost data in Germany from government statistics for UKHD and the nursing home and internal cost information for Charité.

To value capital costs, we used both a top-down and bottom-up approach (Table 1). For UKHD we mostly used market prices, while for Charité we used internal information provided by the purchasing and human resources departments of the hospital. Fix overhead cost information was obtained from different sources including internal purchasing price and open data sources. We estimated the building cost by multiplying the room space used by the commercial rent prices per square meter for UKHD and the nursing home and internal information for Charité. For both hospitals, we computed equipment costs using information on quantities, unit prices, and useful years of life.

### Cost analysis

Data were analyzed in Microsoft Excel 2016. Costs were calculated in Euros using 2021 as the base year. We computed the average cost per test separately for UKHD, Charité, and the nursing home and for the two implementation modalities (i.e., dedicated and non-dedicated staff). Under the non-dedicated staff mode, our calculation assumes that the staff time is used at maximum capacity (100%) across all tasks, not screening alone. Our assumption is based on an understanding that nursing staff divide their time across a number of activities, with Ag-RDTs screening being only one of them. Given that for the dedicated staff mode of implementation, the number of tests performed per day depends not only on the staff time allocated to testing, but also the volume of patients requiring tests, we calculated the average cost per test for the observed capacity use and at three different capacity use assumptions, specifically at 60, 80, 100%. Moreover, we included the RT-PCR confirmation cost for positive cases in the average cost per test. For the calculation of RT-PCR confirmation costs, we used an incidence rate of 0.001, the sensitivity (76.6%) and specificity (99.3%) of the above-mentioned Ag-RDT [10], and the reimbursement rate of RT-PCR in Germany as of February 2021(€50.50) [13].

Related cost projection of Ag-RDTs for hospitals of different service volume was calculated using the estimate of the average costs per test obtained from UKHD and Charité and the expected service volume for each hospital category. Specifically, we used the available open data sources [8, 14, 15] and classified hospitals into three categories based on the number of beds: small (0–300), medium (301–500), and large (500 +). For each hospital category, we used the number of daily in-patient admissions to project the screening costs [8, 14, 15].

### Sensitivity analysis

To assess uncertainty around the average cost per test in each setting, we conducted sensitivity analyses using the minimum and maximum values of the two important and variable cost items: staff time to perform one test (8–16 min) and the unit cost of a test kit (€4 - €6.90).

### Ethics

The costing study received ethical approval (S-802/2020) from the Ethics Commission of the Medical Faculty of Heidelberg University.

## Results

**Fig. 1:**
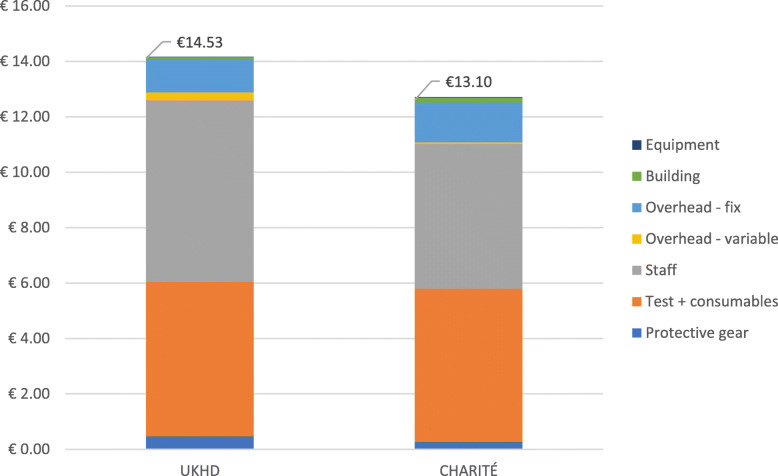
Cost structure per Ag-RDT implemented at UKHD and Charité (non-dedicated mode)

**Fig. 2:**
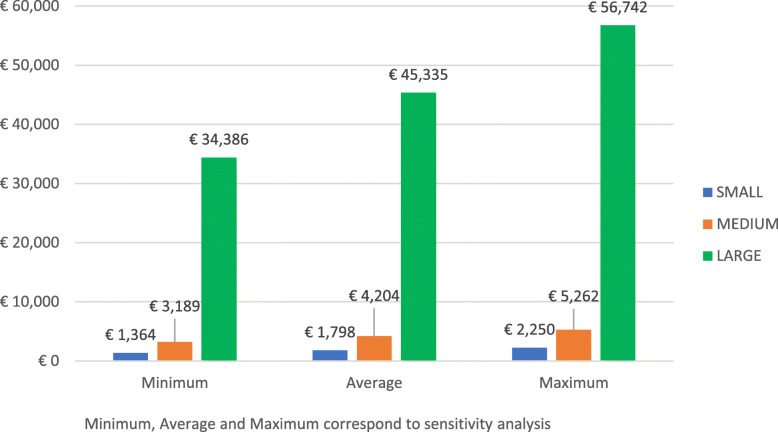
Projection of the daily cost of patient screening in hospitals of different patient volume

In line with our objectives, we first report the cost estimates for the two hospitals (Table 2 and Table 3) and their cost structure (Fig. 1), then the projection of the cost estimates in hospitals of different patient volume (Fig. 2), and finally the cost estimates for the nursing home (Table 4).

**Table 2:** The estimated average cost per test of Ag-RDT implementation in UKHD

SCENARIO	COST ITEMS	DEDICATED MODE				NON-DEDICATED MODE
		CAPACITY OBSERVED	HYPOTHETICAL ASSUMPTION			
			60%	80%	100%	
UKHD	Protective gear	€ 0.68	€ 0.53	€ 0.48	€ 0.46	€ 0.47
	Test + consumables	€ 5.58	€ 5.58	€ 5.58	€ 5.58	€ 5.58
	Staff	€ 20.12	€ 10.95	€ 8.21	€ 6.56	€ 6.56
	Overhead - variable	€ 0.64	€ 0.34	€ 0.29	€ 0.26	€ 0.27
	Overhead - fix	€ 2.37	€ 1.53	€ 1.30	€ 1.18	€ 1.19
	Building	€ 0.31	€ 0.14	€ 0.11	€ 0.08	€ 0.09
	Equipment	€ 0.04	€ 0.02	€ 0.01	€ 0.01	€ 0.01
AVERAGE COST PER TEST		€ 29.73	€ 19.08	€ 15.98	€ 14.14	€ 14.17
(+) PCR CONFIRMATION		€ 30.12	€ 19.47	€ 16.37	€ 14.53	€ 14.56
SENSITIVITY ANALYSIS		LOW	€ 14.40	€ 12.21	€ 11.02	€ 11.06
		HIGH	€ 24.45	€ 20.45	€ 18.30	€ 18.34
Staff time per-test:		Minimum 8 min, average 12 min, maximum 16 min				

*Some variable costs, such as protective gear, are reusable items, thus their quantity does not increase proportionally to the test volume

**Table 3:** The estimated average cost per test of Ag-RDT implementation in Charité Berlin

SCENARIO	COST ITEMS	NON-DEDICATED MODE
		100%
CHARITÉ	Protective gear	€ 0.27
	Test + consumables	€ 5.53
	Staff	€ 5.22
	Overhead variable	€ 0.06
	Overhead fix	€ 1.44
	Building	€ 0.17
	Equipment	€ 0.02
AVERAGE COST PER TEST		€ 12.71
(+) PCR confirmation		€ 13.10
SENSITIVITY ANALYSIS	LOW	€ 9.91
	HIGH	€ 16.28
Staff time per-test:		Minimum 8 min, average 12 min, maximum 16 min

**Table 4:** Estimated average cost per test at the nursing home

SCENARIO	COST ITEMS	NON-DEDICATED MODE
		100%
NURSING HOME	Protective gear	€ 0.90
	Test + consumables	€ 5.58
	Staff	€ 6.56
	Overhead - variable	€ 0.26
	Overhead - fix	€ 1.17
	Building / space	€ 0.28
	Equipment	€ 0.03
AVERAGE COST PER TEST		€ 14.78
(+) PCR confirmation		€ 15.17
SENSITIVITY ANALYSIS	LOW	€ 11.80
	HIGH	€ 18.82
Staff time per-test:		Minimum 8 min, average 12 min, maximum 16 min

We report the average cost per test implemented at UKHD in Table 2 and at Charité in Table 3. For the non-dedicated mode of implementation, where we assume the maximum staff capacity (100%), the estimated average costs per test implemented at UKHD were €14.56 and €13.10 at Charité (including PCR confirmation). The sensitivity analysis for UKHD showed that the average cost per test can vary between €11.06 and €18.34, when implementation time varies from 8 to 16 min and the cost of the above-mentioned test kit varies from €4 to €6.90 (Table 1). The sensitivity analysis for Charité showed a variability of the average cost between €9.91 and €16.28 (Table 3) with the same parameter ranges. For the dedicated mode of implementation, applicable to UKHD only, the estimated average cost per test ranged from €30.12 per test under the observed capacity use to €14.53 (including PCR confirmation) per test under the 100% capacity use assumption (Table 2).

The cost structure is visualized in Fig. 1, which shows that the main cost drivers in hospitals were staff salaries and test kits. Together they accounted for an average of 85% of the total cost, while all recurrent costs accounted for 89% of the total cost (non-dedicated mode).

Figure 2 displays findings from the cost projection in hospitals of different patient volume. Screening of electively admitted patients would cost €1.798, €4.204, €45.335 a day in small, medium, and large hospitals respectively. A sensitivity analysis modifying the two main cost drivers, staff time and cost of tests kits, led to ranges from €1.364 to €2.250 for the small hospital, from €3.189 to €5.262 for the medium hospital, and from €34.386 to €56.742 for the large hospital.

In the nursing home, at maximum capacity utilization, the average costs per test would be €15.17 (including PCR confirmation costs). The sensitivity analysis modifying the two main cost drivers led to ranges from €11.80 to €18.82. As with hospitals, the main cost drivers in the nursing home were staff time and test kits cost, as together they represented 82% of the total cost. Recurrent costs accounted for 90% of total costs.

Our results indicate that in both the dedicated staff mode and the non-dedicated staff mode the estimated cost per test implemented decreases as the maximum capacity utilization is reached.

## Discussion

To the best of our knowledge, this costing study represents the first attempt to estimate the economic costs associated with implementing Ag-RDTs for screening of SARS-CoV-2 in a high-risk setting. Our estimates and projections are intended to provide initial evidence to inform decisions on planning and implementing similar screening strategies in the future. Given limited implementation experience on PoC testing in high-risk settings and related evidence on costing, our findings may also be useful when considering the introduction of test screening programs to prevent outbreaks of other airborne diseases, such as influenza in high-risk settings. Furthermore, the presented methods could be replicated in other high-income settings, with the possibility of estimating the cost of universal screening, including healthcare and administrative staff and visitors.

Our estimates show that the average cost per test implemented by non-dedicated staff at full capacity (100%) was €14.56 in UKHD, €13.10 in Charité and €15.17 in the nursing home. The differences in costs are small and are mainly due to differences in the salaries of nurses and to a lesser extent to differences in the costs obtained for the other items. As can be seen in Fig. 2, in both hospitals staff time and test kits account for approximately 85% of the cost per test implemented. Similarly, the cost per test implemented in the nursing home is highly dependent on the same cost drivers.

According to our estimates, the cost is primarily driven by staff time, therefore the cost of antigen testing is highly dependent on choosing the optimal implementation modality that optimizes staff capacity use. However, this is complex, as it requires detailed planning across different hospital departments and adaptation of patient flow. Estimates of the non-dedicated approach assume that staff works at full capacity, performing tasks pertaining to screening as well as many other nursing tasks, while those of the dedicated approach are based on the observed capacity use of staff time. An important cost difference was seen between clinics that implemented both modalities. Implementing Ag-RDTs at UKHD with dedicated staff (€30.12) was more than twofold than implementing them with non-dedicated staff (€14.56) (Table 1). The difference was driven by the fact that at the time of the study, screening demand, defined in relation to the number of people presenting at UKHD with need to be tested, was not so high as to lead dedicated staff to work at full capacity. This increased the opportunity cost of staff time due to unused time of nurses who were assigned exclusively to do Ag-RDTs testing, possibly leading to efficiency losses in the management of nursing staff time. This points to the importance of adopting the appropriate implementation modality, taking into account the time constraints of health care workers on one side and the expected volume of service on the other, to maximize efficient use of human resources. Considering that the implementation of the dedicated staff mode also implies removing staff temporarily from other nursing tasks, this modality should be considered if, through proper planning, the expected service volume is close to maximum utilization. When this is not feasible, non-dedicated staff mode may represent a preferable option. It is important to note, however, that the time it takes for non-dedicated staff to carry out the tests is time taken away from other nursing activities, capturing the opportunity cost of staff time in performing the tests. Hence, selecting either implementation mode should be a contextual decision, considering a facility capacity, expected testing volume, and overall management feasibility. The second cost driver is the cost of test kits, which have recently dropped rapidly as the quantity demanded increased and a myriad of new tests, although of different accuracy and quality, are now easily available.

Due to daily fluctuations in the number of patients attending clinics, we observed a utilization of the screening capacity use of approximately 45% in clinics that adopted the dedicated-staff approach. Returning to a concept of opportunity cost of staff time outlined earlier, this is because there is downtime throughout the day that is not used to perform other functions, when the staff is assigned exclusively to carry out the tests during their working shift. Dedicated staff mode is more prone to lower efficiency and higher cost in relation to the non-dedicated mode when the planning for the maximum use of staff time is not guaranteed.

We consider that a strength of our study is the evidence on the costs of implementing PoC testing for SARS-CoV-2 detection in high-risk settings, as well as the replicability of the presented methodology.

### Methodological considerations

We need to acknowledge the limited generalizability that arises from our small sample of only two tertiary care hospitals and one nursing home, all located in Germany. The issues of accessibility during the pandemic limited our capacity to enlarge our sample to other high-risk settings that perform screening in Germany and other countries. However, the fact that estimates were relatively stable across settings and across the modifications applied in the sensitivity analysis, such as those related to service volume, suggests that generalizability within the German setting is not as limited as the small study sample would imply. We need to note that by adjusting for different costs for the testing kits, our sensitivity analysis also accounts for different procurement costs since these costs were categorized as fix overheads and calculated as a percentage of the total costs.

Additionally, we need to note that the projections are based exclusively on estimates derived from large tertiary care hospitals even when made for medium- and low-volume facilities. While the sample size is limited, the hospitals are likely to reflect the cost of staff time and implementation complexity for other hospitals. Differences in possible purchase cost across larger volumes were captured with sensitivity analyzes. To account for the potential bias that may arise, we have performed an analysis considering different capacity assumptions. Moreover, due to the risks of conducting observations in nursing homes, the cost estimates were derived exclusively from SOPs and the hospital estimates.

We also need to note two potential limitations of our cost computation, one related to potential costs induced by outbreaks caused by false negative test results and the other related to the cost of having to repeat some tests twice due to the test failure rate. In the first instance, we recognize this as a valid concern, but simply beyond the scope of our analysis, which does not intent to relate cost estimates to transmission parameters. It should be noted, however, that no outbreaks were observed during the implementation period. In the second instance, while in principle, we would have needed to adjust cost estimates to account for test failure rate, we did not because prior evidence revealed that the test implemented in our study, Standard Q COVID-19 Ag SD Biosensor, had no invalid results [16].

## Conclusions

It is important to estimate the volume of tests expected to be performed in the short term in order to choose the appropriate mode of implementation, and thus make the best possible use of available resources. In this way each facility can determine, according to its expected capacity use, the most appropriate screening program for SARS-CoV-2 that can cover not only patients, but also health personnel, administrative staff, and visitors. More evidence is needed on the costs of Ag-RDT implementation in other high-risk, high-income settings, especially in medium and small hospitals, to enhance the evidence-base for decision making.

## Data Availability

All data generated or analysed during this study are included in this published article.

## Abbreviations

Ag-RDT: Antigen-based Rapid Diagnostic Test
RT-PCR: Reverse Transcriptase Polymerase Chain Reaction
PoC: Point of Care
UKHD: Universitätsklinikum Heidelberg
SOP: Standard Operating Procedure
